# Knowledge and Use of Caries Detection Methods among Dental Students and Dental Practitioners in Riyadh, Saudi Arabia

**DOI:** 10.1155/2020/8825890

**Published:** 2020-12-02

**Authors:** Waseem Radwan, Abeer A. AlNasser, Hesah Aloqab, Khalid Al-Saggaf, Nora A. Almuhtab, Bothinah Alnasyan

**Affiliations:** ^1^Department of Restorative Dentistry, College of Dentistry, Riyadh Elm University, Riyadh, Saudi Arabia; ^2^Dental Intern, College of Dentistry, Riyadh Elm University, Riyadh, Saudi Arabia; ^3^Dental Intern, College of Dentistry, Alfarabi Colleges, Riyadh, Saudi Arabia; ^4^Dental Student, College of Dentistry, King Saud University, Riyadh, Saudi Arabia; ^5^Dental Student, College of Dentistry, King Saud Bin Abdulaziz University for Health Sciences, Riyadh, Saudi Arabia

## Abstract

**Aim:**

Accurate detection and diagnosis of dental caries is an integral part of achieving adequate comprehensive dental care. Furthermore, the high prevalence of caries and generally poor oral health in Saudi Arabia is a public health concern. In addition to necessary preventive programs and awareness initiatives, understanding diagnostic practices plays an important role in garnering broad background knowledge regarding the routine diagnostic means utilized by our targeted respondents. Therefore, this study aimed to assess the methods of caries detection among dental students and dental practitioners in Riyadh using a cross-sectional observational study design.

**Methods:**

The sample comprised 496 dental students, interns, postgraduate residents, general dental practitioners, specialists, and consultants from the Riyadh region of Saudi Arabia. A survey was designed to assess caries detection methods, risk assessment practices, and knowledge of advanced diagnostic methods. The responses were correlated with demographic and educational variables. Regression models were used to predict associations.

**Results:**

42.3% and 32.7% use sharp explorers in diagnosing caries always and most of the time, respectively. When conducting caries risk assessment practices, 64.4% was very likely to review the patient's medical history and lifestyle. In terms of knowledge of advanced diagnostic methods, 47.8% know “much” to “very much” about FOTI. The knowledge of advanced caries diagnostic methods and practices of advanced diagnostic methods were significantly positively correlated (*r* = 0.388, *P* < 0.001). Linear regression analysis indicated that higher experience (10+ years) was associated with higher knowledge regarding advanced caries diagnostic methods (*β* = 0.27, *P*=0.009). The mean rank for risk assessment practices was significantly lower in GPs compared to consultants (*P* < 0.05).

**Conclusions:**

The use of traditional and invasive methods of caries detection is prevalent among our respondents, while the usage of advanced diagnostic methods is for the most part low. Therefore, we advocate for more minimally invasive approaches and as such encourage the practice and availability of advanced diagnostic methods.

## 1. Introduction

Oral health is notably associated with overall health, and diseases of the oral cavity have been deemed a public health concern due to their high prevalence and incidence [1, 2]. Dental caries is the most prevalent oral disease [3]; it is defined as a multifactorial disease caused by acid-producing bacteria that attack and damage dental hard tissue. This condition can also be described as a microbial imbalance within the oral cavity in association with factors such as saliva, fluoride exposure, and diet [4, 5]. The prevalence of caries in Saudi Arabia is remarkably high. For example, national statistics showed that the prevalence of dental caries was 71.35% among 15-year-old children in 2019 [6]. Moreover, a cross-regional meta-analysis conducted in 2010 showed a staggeringly high rate of caries among the Saudi population [7]. Similarly, Riyadh, among other locations in Saudi Arabia, suffers from a dental caries epidemic [8, 9].

Caries diagnosis has been defined as identifying or detecting changes in the tooth structure, which are consistent with indicators and factors associated with the disease [10]. It is imperative to acknowledge that the diagnosis of caries is highly dependent on clinicians' skills and experience, among other factors [11]. Caries diagnosis should be rigorous and precise in terms of lesion activity, which subsequently affects the choice of treatment [12]. Moreover, a critical challenge facing clinicians is to detect caries before surgical intervention is needed [4]. Thus, in addition to caries risk assessment and prevention, early detection of caries is essential, as it is a determining factor of patient susceptibility to caries and a critical component when designing an appropriate treatment plan [13]. Caries risk assessment is important for understanding the general oral health status of an individual. Several factors can be measured as indicators of oral health; these factors include bacterial count/type, salivary pH level, dietary habits, and fluoride exposure [14]. These elements are fundamental considerations in clinical examination [15].

Visual examination and radiographic investigation are considered the cornerstones of caries diagnosis [16]. However, methods of caries detection such as sharp explorers are less preferred, especially with the current shift towards minimally invasive modalities and patient-centred approaches in everyday practice [17]. This method can initiate more harm than benefit [15]. Passing a sharp explorer through the pits and fissures of the tooth surface to check for “stickiness” or “catching” is a disputed practice, as it may lead to cavitation in the enamel surface [18].

Moreover, novel advanced diagnostic methods are noninvasive modalities that have been proven to be great adjunctive methods in the diagnosis process, especially in initial lesions where early intervention may halt the disease process allowing more preventive approaches to be implemented [16]. Electronic caries monitor and fiber-optic transillumination have been successfully applied in the diagnosis of caries [19, 20]. Infrared laser fluorescence such as DIAGNOdent is a valid tool in improving diagnostic efficacy [21]. In addition, quantitative light-induced fluorescence offers supplementary chair-side information in diagnosing early enamel lesions [22].

Given the unsatisfactory general oral health status of the population in Saudi Arabia, there is a need for additional preventive and educational dental health programmes [23]. However, the actual usage of caries detection and risk assessment methods by professionals with varying expertise in Saudi Arabia is not well understood. Therefore, we aimed to assess the knowledge and use of different methods to detect dental caries and to perform caries risk assessments among dental students and practitioners in Riyadh, Saudi Arabia.

## 2. Materials and Methods

### 2.1. Ethical Approval

This study was carried out in agreement with the recommendations of the Institutional Review Board of Riyadh Elm University. Ethical approval for the study was obtained from the research centre at Riyadh Elm University (RC/IRB 2019/153).

### 2.2. Survey

This cross-sectional study was conducted using a self-administered survey distributed in June 2019 through two social media outlets (Twitter and WhatsApp); dental students and practitioners were targeted using a convenience sampling method. Participants' consent was obtained before filling out the online survey.

In addition to background and demographic data, the survey assessed three main categories: (1) caries detection methods (i.e., the use of the following: sharp explorer, nonsharp explorer, loupes, ECM, QLF, IRLF, and FOTI), (2) caries risk assessment practices (i.e., caries risk assessment for adults, caries risk assessment for children, evaluation of dietary habits, identifying current fluoride exposure, review medical history and lifestyle choices, and plan the restorative material and technique based on patients caries risk assessment), and (3) knowledge of advanced diagnostic methods (i.e., ECM, QLF, IRLF, and FOTI). The questions were mainly derived from a version of a previously used and validated questionnaire from the College of Dentistry, University of Iowa [24], and were modified and amended to fit the needs of this study.

### 2.3. Study Population

The sample size calculation was performed using the “G^*∗*^Power” sample power calculator [25]. The effective size of 0.1 was used for a population-based survey. It was estimated that, in order to obtain a study power of 0.95, a total of 500 responses would be needed. The study collected a total of 496 responses, resulting in a post hoc sample power of 0.949.

### 2.4. Statistical Analysis

Statistical analysis was performed using R v 3.6.2 [26]. Descriptive statistics are presented as counts and percentages for categorical variables and as means ± standard deviations for continuous variables. Fisher's exact test was used to assess the association between categorical variables, and the two-sided test of equality for column proportions was used to compare the proportion between responses for each question. The Kruskal–Wallis test was used to determine differences in the survey constructs among levels of education and experience.

Linear regression analysis was used to assess factors associated with knowledge regarding the management of dental caries (independent variable). The knowledge score, which is a continuous variable, was used as the dependent variable. Independent variables included demographic characteristics (gender, education, and experience). Linear regression analysis was also used to assess factors associated with knowledge regarding advanced caries diagnostic methods in clinics (dependent variable). Gender, education, and experience were used as independent variables.

## 3. Results

In June 2019, 496 participants, 59.7% (*n* = 296) of whom was male, completed the study survey; the demographic characteristics of the study sample are provided in Table 1. Students represented 23.8% of the study cohort, while postgraduate residents and interns represented 4.84% and 53.6%, respectively. General practitioners (GPs), specialists, and consultants represented 6.85%, 8.06%, and 2.82% of the study cohort, respectively. The amount of experience varied from <5 years (84.9%) to >15 years (2.62%).

The vast majority of participants used sharp explorers (P1) and compressed air drying with illumination (P8) at least some of the time (12.1% and 15.9%, respectively); 75% and 45.6%, respectively, used these methods most of the time or always in diagnosing caries (Table 2). It was observed that a significant majority of the sample surveyed reported never using advanced caries diagnostic methods (*P* < 0.05).

Regarding the caries risk assessment category of the survey, more than half of the participants reported that they were likely or very likely to use each of the caries risk assessment practices evaluated (Table 3). Specifically, the majority of the included participants reported that they were very likely (64.4%) or likely (26.1%) to review the medical history and lifestyle of their patients when assessing caries risk practices. Moreover, the respondents performed caries risk assessment for adult patients (47.1% and 32.6% replied to this question with very likely and likely, respectively) and planned restorative materials and techniques based on the risk assessment (53.2% and 31.9% replied to this question with likely and likely, respectively).

Responses for the remaining four questions related to knowledge of advanced caries detection methods are shown in Table 4. Knowledge regarding the electronic caries monitor (ECM) varied from none (18.1%) to very much (13.7%), and similar patterns were observed for knowledge regarding QLF, IRLF, and FOTI.

The results from the linear regression analysis (Table 5) showed that more experience (10+ years) was associated with more knowledge regarding advanced caries diagnostic methods (*β* = 0.27; *P*=0.009). Similarly, education was also associated with knowledge regarding advanced caries diagnostic methods; the average knowledge score was 0.41 points lower for GPs than among students, interns, and residents (*β* = −0.41; *P* < 0.001). Gender was not associated with knowledge regarding advanced caries diagnostic methods (*ββ* = −0.13; *P*=0.184).

Cronbach's *α* for the three included scales was >0.7 (Table 6), which is considered an appropriate indicator of good reliability. The correlation matrix showed that there was a significant positive correlation between knowledge and practice of advanced caries diagnostic methods (*r* = 0.388; *P* < 0.001), indicating that greater knowledge regarding the new diagnostic methods was associated with more positive practices. Similarly, knowledge of advanced caries diagnostic methods and the performance of caries risk assessment methods were significantly positively correlated (*r* = 0.191; *P* < 0.001). The correlation between the practice of caries detection methods and the assessment of caries risk was not significant (*r* = −0.038; *P* > 0.05).

The 16 included items were categorized based on item loading >0.7 into three factors, caries detection method practices, knowledge regarding advanced caries detection methods, and caries risk assessment practices (Table 6), indicating that the convergent validity assumption was met.

We next identified significant differences in the practice of caries detection methods (*P* < 0.001), the assessment of caries risk (*P* < 0.05), and knowledge regarding advanced caries diagnostic methods (*P* < 0.001) across participants with different levels of education with the Kruskal–Wallis test (Table 7). Post hoc pairwise comparisons indicated that the mean rank for the practice of caries detection methods was significantly lower in interns than in postgraduate residents, specialists, or consultants (*P* < 0.05), indicating that interns, who typically have less education, practiced the various caries detection methods less often. The practice of caries detection methods was not significantly different between any of the remaining pairs evaluated. The mean rank for caries risk assessment practices was significantly lower in GPs than in consultants (*P* < 0.05), indicating that GPs, who typically have less education than consultants, practiced the evaluated caries risk assessment methods less often. The caries risk assessment practices were not significantly different between any of the remaining pairs. Knowledge regarding advanced caries detection methods was significantly lower in interns than in students, postgraduate residents, or specialists (*P* < 0.05).

## 4. Discussion

Identifying caries detection methods practiced by our respondents will assist in understanding the diagnostic process involved in everyday practice. This is particularly crucial in the prevention and control of dental caries, especially in its early reversible stages. In this paper, a substantial quantity of data has been gathered regarding the knowledge and use of caries detection and caries risk assessment methods.

The majority of respondents indicated the regular use of both sharp explorers and compressed air drying for caries diagnosis. This is consistent with the current findings, and pertinent research conducted in Turkey also indicated that the majority of private practice dentists use sharp explorers [27]. It was found that such an approach contravenes the preventive and conservative approach of “minimally invasive dentistry,” which emphasizes retaining as much of the natural tooth structure as possible [17]. The latter approach is also considered contrary to G.V. Black's renowned “extension for prevention” approach [28]. Ball-ended explorers, otherwise known as nonsharp or blunt dental explorers, have been shown to cause less damage than sharp explorers during the examination of a tooth [29]. However, among our sample, there was unsatisfactory use of ball-ended explorers, which may be due to decreased availability in dental clinics; further investigation is needed to determine the reason that this tool is underutilized.

On the other hand, the use of compressed air drying, which was also reported to be regularly used by the majority of respondents, has been shown to be reliable in the early detection of caries [30, 31]. However, this method has been reported to have high specificity but low sensitivity in caries diagnosis compared to other diagnostic methods [31].

There is evidence to support the use of magnification loupes in the diagnosis of caries, especially in minimally invasive approaches [17]; yet, our findings imply low adoption of this tool. Similarly, the utilization of magnification loupes in relation to a minimally invasive approach adopted by dentists in Riyadh and Al-Kharj cities was deemed statistically nonsignificant [32].

In terms of caries risk (i.e., the chance of developing new lesions) [15], medical history and lifestyle choices review were reported to be used by the study participants. This suggests that the practitioners likely have good history-taking skills and regularly perform a thorough investigation prior to treatment, which is an essential practice for identifying any pathology or disease that may require some adjustment or modification of the subsequent dental treatment.

Moreover, lifestyle habits have a profound effect on general and oral health. In a study conducted in 2019, females with a healthy lifestyle were found to have more favourable oral health habits than their male counterparts [33]. In the current study, more than 80% of respondents, especially consultants, reported that they were likely or very likely to plan restorative treatment based on the patient's risk assessment and conduct a caries risk assessment in adults. This is especially important in high-risk cases where the need for rehabilitation using temporary restorations and extensive preventive measures is advocated to stabilize the condition for long-standing results [34]. Similarly, Elagra et al. concluded that general dentists preferred to conduct restorative treatment over preventive modalities in high-risk patients [35].

Notably, nearly 40% of responders reported that they were unlikely or very unlikely to evaluate patients' fluoride exposure. However, fluoride levels in drinking water in Saudi Arabia correlate significantly with caries incidence and the prevalence of dental fluorosis [36], and therefore, fluoride exposure assessment should be a part of routine patient evaluation.

It was observed that the most known advanced caries detection device was FOTI, followed by QLF. These enhanced visual devices have shown to be accurate and noninvasive caries detection methods [16]. However, the usage of these devices was generally low. The advanced caries detection methods evaluated, namely, ECM, QLF, IRLF and FOTI, were all seldom used when diagnosing caries. Therefore, while respondents indicated some knowledge of advanced caries detection methods, the reported utilization was even lower. Variable levels in the knowledge of advanced dental caries diagnosis methods among dental students as well as dental practitioners were noted. This is consistent with studies from across the world that have shown that the practice of dentistry has not kept pace with the rapid advances made in the principles of dental caries diagnosis [37, 38]. Correspondingly, in a study conducted in 2011, it was found that low use of FOTI among dentists in out-patient clinics was observed [38]. The justification may be due to the decreased availability and accessibility of such tools, and further exploration of this matter is necessary. Furthermore, no single system can be used to detect caries on all surfaces, and several methods should be used to detect caries on a tooth with multiple surface lesions [39]. Additionally, the utilization of these advanced methods in everyday clinical use is disputed; nevertheless, the utility of FOTI should not be underestimated. Studies have noted that FOTI detects more occlusal and interproximal caries than other methods [19], and it is recommended as an adjunct detection tool that should be included in daily practice [40].

As previously mentioned, an association between higher education levels and a greater understanding of advanced diagnostic measures was observed. The positive correlation between knowledge and usage of advanced caries detection methods suggests that educating practitioners could increase the adoption of these techniques in the clinic. Moreover, the level of knowledge regarding these advanced caries detection methods was significantly lower in interns than in students, postgraduate residents, and specialists. The basis for such outcomes cannot be elaborated upon, and further investigation is required.

## 5. Limitations

The reliability and generalizability of these data are assumed to be limited due to the online distribution of the survey. Because of this methodological drawback, the sample population is not representative of all dental practitioners and students in Riyadh. Language may also be considered a limitation, as some technical terms/or devices are not generally known.

## 6. Conclusion

Despite the limitations of this study, it can be concluded that there is some knowledge and little usage of advanced caries diagnostic methods among both dental practitioners and dental students in Riyadh. Although there seems to be an acceptable awareness and practice of caries risk assessment methods in the planning of restorative treatment, additional education regarding advanced methods of caries diagnosis is needed for practitioners and students. Moreover, conventional methods such as using sharp explorers are still commonly used, despite evidence that they are harmful to patients. Additional education in this regard is also needed.

The prevailing attitudes and trends of the dental community as of late are shifting towards a minimally invasive and preventive approach. Considering that Saudi Arabia is currently experiencing an oral health epidemic [41], the additional education suggested may address some of the gaps identified in this study to help support caries diagnosis and tooth preservation. We recommend conducting similar studies on a national scale, to help understand and assess the caries detection methods in such a liable population.

## Figures and Tables

**Table 1:** Demographic characteristics of the study sample.

	*N* = 496
Gender	
Female	200 (40.3%)
Male	296 (59.7%)

Education	
Student	118 (23.8%)
Intern	266 (53.6%)
Postgraduate resident	24 (4.84%)
GP	34 (6.85%)
Specialist	40 (8.06%)
Consultant	14 (2.82%)

Experience	
<5 years	421 (84.9%)
5–9 years	47 (9.48%)
10–15 years	15 (3.02%)
>15 years	13 (2.62%)

**Table 2:** Frequency of use of caries detection methods.

Method	Never or rarely (0–9%)	Sometimes (10–49%)	Often (50–74%)	Most of the time (75–99%)	Always (100%)
P1-use of sharp explorer	35 (7.06%)^a^	29 (5.85%)^a^	60 (12.1%)^a^	162 (32.7%)^b^	210 (42.3%)^b^
P2-use of explorer that is not sharp	243 (49.0%)^a^	90 (18.1%)^b^	44 (8.87%)^b^	60 (12.1%)^b^	59 (11.9%)^b^
P3-magnification (e.g., loupes)	298 (60.1%)^a^	64 (12.9%)^b^	48 (9.68%)^b^	47 (9.48%)^b^	39 (7.86%)^b^
P4-ECM (electrical caries monitor)	385 (77.6%)^a^	35 (7.06%)^b^	23 (4.64%)^b^	29 (5.85%)^b^	24 (4.84%)^b^
P5-QLF (quantitate light-induced fluorescence)	374 (75.4%)^a^	39 (7.86%)^b^	30 (6.05%)^b^	29 (5.85%)^b^	24 (4.84%)^b^
P6-IRLF (infrared laser fluorescence)	388 (78.2%)^a^	33 (6.65%)^b^	25 (5.04%)^b^	32 (6.45%)^b^	18 (3.63%)^b^
P7-FOTI (fiber-optic transillumination)	338 (68.1%)^a^	60 (12.1%)^b^	38 (7.66%)^b^	40 (8.06%)^b^	20 (4.03%)^b^
P8-compressed air drying with illumination	124 (25.0%)^a^	67 (13.5%)^a^	79 (15.9%)^a^	103 (20.8%)^a^	123 (24.8%)^a^

^a,b^Values in the same row and subtable not sharing the same subscript are significantly different at *P* < 0.05 in the two-sided test of equality for column proportions. Cells with no subscript are not included in the test. Tests assume equal variances. Tests are adjusted for all pairwise comparisons within a row of each innermost subtables using the Bonferroni correction.

**Table 3:** Caries risk assessment practices.

Method	Very unlikely	Unlikely	Likely	Very likely
A1-caries risk assessment for adult patients	28 (5.81%)^a^	70 (14.5%)^a^	157 (32.6%)^b^	227 (47.1%)^b^
A2-caries risk assessment for children	41 (8.54%)^a^	89 (18.5%)^a^	140 (29.2%)^a^	210 (43.8%)^b^
A3-evaluate the patients' dietary habits	29 (6.02%)^a^	83 (17.2%)^a^	215 (44.6%)^b^	155 (32.2%)^b^
A4-identify current exposures to fluoride	52 (10.9%)^a^	125 (26.2%)^a^	181 (37.9%)^b^	120 (25.1%)^a^
A5-review medical history and lifestyle	10 (2.02%)^a^	37 (7.49%)^a^	129 (26.1%)^b^	318 (64.4%)^c^
A6-plan restorative materials and techniques based on the patients' caries risk assessment	20 (4.09%)^a^	53 (10.8%)^a^	156 (31.9%)^b^	260 (53.2%)^b^

^a, b^Values in the same row and subtable not sharing the same subscript are significantly different at *P* < 0.05 in the two-sided test of equality for column proportions. Cells with no subscript are not included in the test. Tests assume equal variances. Tests are adjusted for all pairwise comparisons within a row of each innermost subtable using the Bonferroni correction.

**Table 4:** Reported knowledge of advanced diagnostic methods.

Method	None	Little	Some	Much	Very much
ECM (electrical caries monitor)	90 (18.1%)^a^	128 (25.8%)^a^	111 (22.4%)^a^	99 (20.0%)^a^	68 (13.7%)^a^
QLF (quantitative light-induced fluorescence)	80 (16.1%)^a^	118 (23.8%)^a^	98 (19.8%)^a^	119 (24.0%)^a^	81 (16.3%)^a^
IRLF (infrared laser fluorescence)	89 (17.9%)^a^	131 (26.4%)^a^	103 (20.8%)^a^	114 (23.0%)^a^	59 (11.9%)^a^
FOTI (fiber-optic transillumination)	69 (13.9%)^a^	105 (21.2%)^a^	85 (17.1%)^a^	127 (25.6%)^a^	110 (22.2%)^a^

a,bValues in the same row and subtables not sharing the same subscript are significantly different at *P* < 0.05 in the two-sided test of equality for column proportions. Cells with no subscripts are not included in the test. Tests assume equal variances. Tests are adjusted for all pairwise comparisons within a row of each innermost subtable using the Bonferroni correction.

**Table 5:** Association of demographic characteristics with knowledge regarding advanced caries diagnostic methods in clinics.

Predictors	Estimates	CI	*p*
Intercept	0.27	0.08–0.47	0.005
Experience: 1–4 years	Ref		
Experience: 5–9 years	0.14	−0.23–0.52	0.454
Experience: 10+ years	0.64	0.16–1.13	0.009
Gender: females	Ref		
Gender: male	−0.13	−0.32–0.06	0.184
Education: student/intern/resident	Ref		
Education: GP	−0.41	−0.62–−0.19	<0.001
Education: specialist/consultant	0.00	−0.41–0.42	0.988
Ref: referent category			

**Table 6:** Correlation matrix demonstrating caries detection methods, knowledge of advanced diagnostic methods, and caries risk assessment practices.

Factor	Caries detection methods	Knowledge regarding advanced caries diagnostic methods	Risk assessment practices
Α	0.878	0.867	0.937
Caries detection methods	1.000		
Knowledge regarding advanced caries diagnostic methods	0.388^*∗∗∗*^	1.000	
Risk assessment practices	−0.038	0.191^*∗∗∗*^	1.000

**Table 7:** Comparison of the knowledge and use of caries detection and caries risk assessment methods across levels of education.

	Student	Postgraduate resident	Intern	GP	Specialist	Consultant	*p*
	*N* = 118	*N* = 24	*N* = 266	*N* = 34	*N* = 40	*N* = 14	
*P*	1.50ab (1.00; 2.29)	1.50a (1.33; 1.67)	1.17b (1.00; 2.00)	1.33ab (1.17; 1.96)	1.50a (1.17; 2.00)	1.67a (1.38; 2.21)	<0.001
*A*	3.17ab (2.83; 3.67)	3.27ab (2.67; 3.70)	3.33ab (2.67; 3.83)	3.00b (2.52; 3.33)	3.33ab (2.83; 3.50)	3.75a (3.33; 4.00)	0.031
*K*	3.50b (2.06; 4.00)	3.88b (2.88; 4.25)	2.62a (2.00; 3.50)	3.12ab (2.06; 3.75)	4.00b (2.94; 4.50)	4.00ab (2.12; 5.00)	<0.001

*P*, caries detection methods; *A*, caries risk assessment practices; *K*, knowledge regarding advanced caries diagnostic methods. Results were summarized using the median and interquartile range (IQR). Statistical analysis was performed using the Kruskal–Wallis test. Scores were calculated by averaging the responses that correspond to each factor.

## Data Availability

The data used to support the findings of this study are included within the article.
